# Persistent Enhancement of Hippocampal Network Connectivity by Parietal rTMS Is Reproducible

**DOI:** 10.1523/ENEURO.0129-19.2019

**Published:** 2019-10-15

**Authors:** Michael Freedberg, Jack A. Reeves, Andrew C. Toader, Molly S. Hermiller, Joel L. Voss, Eric M. Wassermann

**Affiliations:** 1 National Institute of Neurological Disorders and Stroke, Bethesda, MD 20892; 2 Henry M. Jackson Foundation for the Advancement of Military Medicine, Bethesda, MD 20817; 3 Northwestern University Interdepartmental Neuroscience Program, Northwestern University, Chicago, Illinois; 4 Ken and Ruth Davee Department of Neurology, Feinberg School of Medicine, Northwestern University, Chicago, Illinois 60611; 5 Department of Medical Social Sciences, Feinberg School of Medicine, Northwestern University, Chicago, Illinois 60611; 6 Northwestern University Interdepartmental Neuroscience Program, Northwestern University, Chicago, Illinois 60611

**Keywords:** fMRI, functional connectivity, hippocampus, TMS

## Abstract

Wang et al. (2014) found that that five daily sessions of repetitive transcranial magnetic stimulation (rTMS) of the posterior parietal cortex (PPC) significantly increased functional connectivity (FC) in a network centered on the hippocampus, and caused a correlated increase in memory performance. However, this finding has not been reproduced independently and the requirement for five sessions has not been validated. We aimed to reproduce the imaging results of this experiment, focusing on hippocampal FC changes and using fewer days of rTMS. We measured resting state FC before and after three (*N* = 9) or four (*N* = 6) consecutive daily PPC rTMS sessions, using similar delivery parameter settings as Wang et al. (2014). Eight subjects received 3 d of rTMS delivered to the vertex as a control. We employed whole-brain and hypothesis-based statistical approaches to test for hippocampal FC changes. Additionally, we calculated FC in 17 brain networks to determine whether the topographic pattern of FC change was similar between studies. We did not include behavioral testing in this study. PPC, but not vertex, rTMS caused significant changes in hippocampal FC to the same regions as in the previous study. Brain-wide changes in hippocampal FC significantly exceeded changes in global connectedness, indicating that the effect of PPC rTMS was specific to the hippocampal network. Baseline hippocampal FC, measured before receiving stimulation, predicted the degree of rTMS-induced hippocampal FC as in the previous study. These findings reproduce the imaging findings of Wang et al. (2014) and show that FC enhancement can occur after only three to four sessions of PPC rTMS.

## Significance Statement

One of the most striking recent findings in the area of neuromodulation is that of Wang et al. (2014), who reported that posterior parietal cortex (PPC) stimulation increased functional connectivity (FC) in a network related to declarative memory and centered on the hippocampus, a result with great potential experimental and clinical utility. We used a similar paradigm, with shorter treatment duration and reproduced the effects on connectivity, including specificity for the hippocampal network and dependence on the magnitude of baseline hippocampal connectivity. These results confirm and extend the initial finding and validate the technical approach.

## Introduction

Enhancing memory in patients and healthy individuals is a potential application of repetitive transcranial magnetic stimulation (rTMS). Network connectivity modulation with non-invasive brain stimulation has been studied mostly in motor and procedural learning networks (Muellbacher et al., 2002; Baraduc et al., 2004; Wilkinson et al., 2010, 2015; Hotermans et al., 2008; Rosenthal et al., 2009; Iezzi et al., 2010; Teo et al., 2011) and the effects have not been shown to last longer than minutes or hours (Thut and Pascual-leone, 2010). The declarative memory system, on the other hand, has been less explored with rTMS, despite the fact that declarative memory deficits are among the most common and debilitating problems in neurology (Vakil, 2005; Nestor et al., 2006). Wang et al. (2014) increased declarative memory and resting hippocampal network functional connectivity (FC) by delivering multiple-session rTMS to individualized targets in the posterior parietal cortex (PPC), which is connected with the hippocampus via the retrosplenial and paraphippocampal cortices (Mesulam et al., 1977; Cavada and Goldman-Rakic, 1989). The FC increase and memory improvement persisted for 24 h after the final rTMS session and, with reduced strength, for up to approximately two weeks (Wang et al., 2014; Wang and Voss, 2015).

The Wang et al. (2014) findings are a dramatic demonstration of physiologic engagement of a specific brain target with correlated behavioral improvement and are, in this respect, unique in the noninvasive neuromodulation field. However, concern has grown over the rate of false positives in functional neuroimaging (Poldrack et al., 2017) and noninvasive neuromodulation (Nahas et al., 2008; Héroux et al., 2015), resulting in calls for reproduction of results. For example, Héroux et al. (2015) found that only between 45% and 60% of experienced researchers were able to reproduce a rTMS effect.

In this study, we used a similar paradigm to that of Wang et al. (2014), with identical targeting procedures and stimulation parameter values, but with fewer stimulation sessions and without memory testing. We also preprocessed the data somewhat differently and used vertex stimulation, instead of subthreshold or motor stimulation, as our control condition. Although the original researchers collaborated on this study and shared unpublished data and techniques with us, all data collection, implementation, and analysis were performed independently.

## Materials and Methods

### Subjects

Twenty-three healthy adults (nine females; age = 19–31 years), free of neurologic or psychiatric disorders or medications acting on the central nervous system, participated in the study. Fifteen received active rTMS delivered to the PPC and eight underwent a control procedure with identical stimulation applied to the vertex. All subjects reported being right-handed and passed screening for contraindications to TMS (Rossi et al., 2009) and MRI. Written informed consent was obtained and the study was approved by the local Institutional Review Board.

### Procedures

All subjects underwent, in order, baseline scanning, three or four consecutive daily rTMS sessions, and a post-rTMS scan. Baseline scanning included an anatomical localizer, structural scan (for functional scan co-localization with anatomy, and neuro-navigation), a single resting state scan, and diffusion tensor imaging (not reported here). Nine subjects received three consecutive daily sessions of rTMS delivered to the PPC, six received four daily PPC sessions, and eight received three daily sessions of identical rTMS delivered to the vertex (see rTMS, below). The interval between rTMS sessions was ∼24 h.

Twelve of our PPC subjects participated in a separate study to find the minimum number of days required to produce a conservative criterion change in hippocampal FC. We found no measurable difference in response between subjects receiving 3 and 4 d of rTMS (*W* = 36, *p* = 0.327, 95% CI [–0.074333, 0.210993]), so all were included here. The number of stimulation sessions in this study differed from Wang et al. (2014), who delivered stimulation on five consecutive days.

Unlike Wang et al. (2014), we used rTMS at the vertex, which produces auditory and somatosensory stimulation, but no significant changes in FC (Jung et al., 2016), as our control condition (see Discussion). Subjects underwent the first rTMS session within 36 h of baseline scanning. The second MRI session occurred on the day after the final rTMS session and within 3 h of the time of day of the first scanning session. Subjects were blind to the specific intent of the study and the stimulation condition.

### fMRI acquisition and preprocessing

MRI was performed on a Siemen’s Magnetom 3T scanner using a 16-channel head coil with foam padding to prevent head movement. Subjects were fitted with earplugs and supplied with headphones to protect hearing. During resting scans, subjects were instructed to lie still with their eyes open.

Blood oxygen level-dependent (BOLD) data were recorded with a T2*-weighted gradient-echoplanar imaging sequence (EPI: TR = 2000 ms, TE = 27 ms, flip angle = 90°, 36 transversal contiguous interleaved slices per volume, 3.0 slice thickness, FOV 22 × 22 cm, matrix size 64 × 64, voxel size = 3.4 × 3.4 × 3.0 mm; scan length ∼6.8 min). We acquired structural images with a magnetization-prepared rapid gradient echo sequence (MPRAGE; TR = 2530 ms, TE = 3.03 ms, 176 slices per volume, 1-mm thickness, FOV = 25.6 × 25.6 cm^2^, 256 × 256 acquisition matrix, voxel size = 1.0 mm isotropic, 206 volumes, 6.83 min).

We processed the images with analysis of functional images (AFNI; Cox, 1996; RRID:SCR_005927) software. The first five volumes of 206 were removed to ensure that magnetization was stabilized. Preprocessing included motion correction, slice-timing correction to the first slice, functional/structural affine co-registration to Talairach space (TT_N27; Talairach and Tournoux, 1988), resampling to 2.0 mm isotropic voxel resolution, spatial smoothing using a 4-mm full-width half maximum (FWHM) Gaussian kernel, and linear detrending. We then scaled each voxel time series to a mean of 100, with a range of 0–200 and regressed head motion from each voxel time series using the mean and derivatives of six parameter estimates (pitch, roll, yaw, and rotation around each axis). Unlike Wang et al. (2014), we did not bandpass filter our data because test-retest reliability increases as the high pass cutoff is raised and even eliminated (Shirer et al., 2015). However, we achieved a high-pass filter via linear detrending using a 2nd or 3rd order polynomial, depending on the subject. We used spatial smoothing, which was omitted by Wang et al. (2014). Finally, frames which included movement displacement >0.3 mm were censored before statistical analysis to prevent inflated correlations (Power et al., 2012). We used a threshold of 0.3 mm of average head displacement across all frames, including censored ones, during any scan to exclude subjects (one subject).

We reprocessed and reanalyzed data from Wang et al. (2014), which were acquired on a Siemens 3T TIM Trio with a 32-channel head coil. Structural (MPRAGE T1-weighted scans, TR = 2400 ms, TE = 3.16 ms, voxel size = 1 mm^3^, FOV = 25.6 cm, flip angle = 8°, 176 sagittal slices) and functional whole-brain BOLD EPI (TR = 2500 ms, TE = 20 ms, voxel size = 1.72 × 1.72 × 3 mm^3^, FOV = 22 cm, flip angle = 80°, 244 volumes, 10.2 min). We handled them identically to our own data, but resampled to 1.5-mm isotropic voxel resolution.

### rTMS targeting

**Figure 1.**
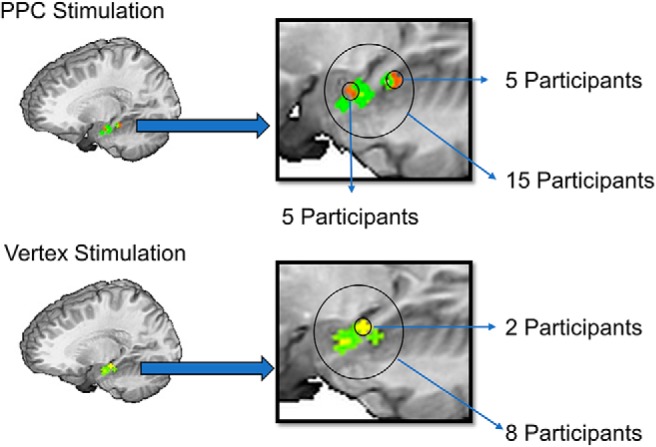
Seed locations from PPC (top; *N* = 15) and vertex groups (bottom; *N* = 8).

We based our targeting procedure on Wang et al. (2014) who chose the PPC subregion that was maximally connected to the hippocampus in each subject. They searched the anterior/middle hippocampus for the voxel with maximal FC to the PPC and chose the PPC location where this FC was strongest as the stimulation target. We applied a similar technique. For subjects receiving PPC stimulation, we guided rTMS to the PPC location with maximum FC to a seed location in the hippocampus. In each subject, the PPC target search volume was a sphere of 15-mm radius, cut to exclude non-brain voxels, around Talairach location *x* = –47, *y* = –68, *z* = +36, which included the supramarginal and angular gyri. The search for the hippocampal seed voxel involved two approaches, both employing automated scripts. For the first approach (12 subjects), we chose the maximally connected hippocampal voxel from six preselected locations along the longitudinal aspect of the hippocampus in Talairach–Tournoux space (seed 1: *x* = –26, *y* = –10, *z* = –17; seed 2: *x* = –22, *y* = –16, *z* = –13; seed 3: *x* = –30, *y* = –17, *z* = –14; seed 4: *x* = –30, *y* = –22, *z* = –12; seed 5: *x* = –30, *y* = –27, *z* = –9; seed 6: *x* = –30, y – 32, *z* = –6). This deviated from the seeding procedure of Wang et al. (2014), who sampled only from the anterior/middle hippocampus. In the second approach (three subjects), we selected the maximally connected one of 97 preselected voxels in the anterior hippocampus. These included hippocampal voxels within 15 mm of the Talairach coordinates identified in Wang et al. (2014; *x* = –24, *y* = –18, *z* = –18). This approach was intended to provide wider sampling within the hippocampus. Figure 1 illustrates the seed locations for each subject. In both approaches, we created a 3-mm radius sphere around the coordinates of each voxel in the search and computed an average time series using the voxels in that sphere. We then searched the PPC sphere for the voxel with maximum correlation with the hippocampal seed, marked its location in standard space, and then back-transformed the location into subject space using the inverse matrix of the original affine transformation. Next, this location was transformed into a 3-mm radius sphere and overlaid on the subject’s structural MRI for rTMS targeting with the Brainsight frameless stereotaxic system. For the PPC target, a stimulation trajectory was created in Brainsight, so that the plane of the coil was tangential to the scalp and the induced current field was oriented perpendicular to the long axis of the gyrus containing the stimulation target. For control stimulation, we located the vertex using the 10-20 International system (Steinmetz et al., 1989), and held the coil tangential to the scalp with the junction of the coil lobes in the sagittal axis.

### rTMS

TMS was delivered with a MagStim Rapid^2^ stimulator through a Double Airfilm coil. Wang et al. used a Nexstim eXimia NBS 4.3 air-cooled, MRI-guided system and a 70-mm figure eight coil. rTMS intensity was referenced to the individual motor evoked potential threshold, which was determined in the current experiment immediately before the first rTMS session using the TMS Motor Threshold Assessment Tool (MTAT 2.0; http://www.clinicalresearcher.org/software.htm). Stimulation parameter settings for PPC and vertex stimulation were identical to those of Wang et al. (2014), i.e., 2-s trains at 20 Hz (40 pulses per train) with an intertrain interval of 28 s, at 100% of resting motor threshold (RMT). There were 40 trains, 1600 pulses, and a duration of 20 min per session.

### FC calculations and voxel-wise analysis

For all hippocampal FC analyses, we conducted the following steps: Preprocessed data from the prestimulation and poststimulation resting state scans were seeded at the hippocampal location maximally connected with the PPC in the prestimulation scan, the area found for rTMS targeting. We created a 3-mm radius sphere around this location and averaged the BOLD time series of all voxels within it to derive a single hippocampal time series. Pearson’s *r* values were then computed for the correlation between this time series and that from every voxel in the rest of the brain. Finally, all *r* values were r-to-z Fisher transformed to form a final connectivity metric [z_(r)_] across voxels for each scan.

### Whole brain changes in hippocampal network FC and comparison to Wang et al. (2014)

To identify areas where PPC rTMS caused significant changes in hippocampal FC, and to see whether they were in the same areas reported by Wang et al. (2014), z_(r)_ values for each subject and time point, prestimulation and poststimulation, were fed into AFNI’s *3dttest++* command for comparison. A group mask excluded ventricles and white matter. The results were false discovery rate (FDR) corrected at *q* = 0.05. We applied Bonferroni corrected *post hoc* tests to significant clusters in regions where Wang et al. (2014) reported significantly greater hippocampal FC increases with active compared to sham rTMS. These included the precuneus/retrosplenial, fusiform, lateral parietal, and superior parietal areas (α = 0.05/4 = 0.0125). Wilcox rank-sum testing was used for significance testing since these data were non-normally distributed.

### Hypothesis-based comparison to the Wang et al. (2014) results

We performed this analysis to see whether PPC rTMS in the current study caused significant increases in hippocampal FC within a mask of regions showing significant hippocampal FC change in the reanalyzed data of Wang et al. (2014). To determine this region-of-interest, we searched for areas of the posterior left hemisphere that showed a significant increase in hippocampal FC after active rTMS, relative to sham, and calculated z_(r)_ values as described above. For each subject in the data set of Wang et al. (2014), the prestimulation correlation map was subtracted from poststimulation map, and the pre-sham map from the post-sham map. We then fed these subtractions into AFNI’s *3dttest++* command for contrast. Like Wang et al. (2014), we applied a cluster size threshold of 290 voxels and identified a cluster encompassing the left precuneus and medial occipital lobe (left precuneus/occipital cortex; LPOC). We created a mask from these regions by applying the *3dclust* command in AFNI and resampling the mask to the geometry of our own dataset (2-mm isotropic voxels). To account for variability across subjects, we dilated the mask by three voxels while restricting voxels to the left hemisphere. The pattern of results did not change based on the dilation of the mask. Finally, using the present data, we calculated prestimulation and poststimulation hippocampal FC in these regions and contrasted the resulting prestimulation and poststimulation z_(r)_ values using a Wilcox rank-sum test, to look for a significant PPC rTMS-related change in FC between the hippocampus and the LPOC region, like that reported by Wang et al. (2014).

We also calculated the change in hippocampus-LPOC FC with vertex rTMS. Here, we used the hippocampal seed that was maximally connected with the PPC target at baseline, and the same automated script applied to the PPC subjects, to avoid potential bias in the selection of seeds. To determine whether changes in hippocampal FC with the LPOC mask were specific to PPC stimulation, we compared the rTMS-related change in hippocampus-LPOC FC between groups with the Mann–Whitney test. Additionally, to determine whether our results were affected by differences in sample size between groups, we performed a permutation test using matched sample sizes. This was performed by subtracting the mean FC change of the vertex group from the mean FC change in eight subjects randomly selected from the PPC group. This was performed 1000 times to form a distribution of possible outcomes, which we then compared to the observed mean difference.

### Specificity analysis

To gauge the specificity of the Wang et al. (2014) effect on FC, we compared the changes in hippocampal FC and global connectedness (GC) occurring in the LPOC mask (LPOC-GC) with PPC rTMS. To calculate LPOC-GC, we found Pearson’s *r* values for each voxel in the brain for the correlation of its time series with those of every other voxel. Next, we calculated the mean of all of the *r* values for each voxel within the LPOC mask (Gotts et al., 2012). The mean *r* values were then r-to-z Fisher transformed to create a GC value for each voxel. Finally, all voxel GC values in the LPOC mask were averaged.

As an additional control, we calculated the change in FC between the left dorsolateral prefrontal cortex (DLPFC) and the LPOC mask with the expectation that PPC stimulation would not significantly enhance FC between these regions. We created the DLPFC seed by forming a 3-mm radius sphere around Talairach and Tournoux location *x* = –41, *y* = 44, *z* = 5, a peak area of activation found during procedural learning (Poldrack et al., 2001). The mean time series from this sphere was then compared with that from every voxel in the LPOC mask. Finally, we took the mean of all r-to-z transformed values in the LPOC mask. Wilcox rank-sum tests were performed to determine whether the hippocampal-LPOC FC change differed significantly from the DLPFC-LPOC FC and LPOC-GC changes.

### Comparison of topographic changes

We assessed the topographic pattern of hippocampal FC changes from PPC stimulation by calculating the change in hippocampal FC with 17 segregated networks (Yeo et al., 2011) using AFNI’s *3dBrickStat* command. We also calculated within-network GC for this analysis using the time series of all voxels in each of the 17 networks. GC for each network was calculated as the mean z_(r)_ value across all voxels in that network. We then compared the hippocampal FC and GC changes. The same steps were performed using the pre- to post-active stimulation data from Wang et al. (2014). We performed hippocampal-FC to GC comparisons for each study with one-sample, two-tailed, *t* tests, since these data were normally distributed.

Finally, to test the hypothesis that the magnitude of hippocampal FC changes across networks were correlated across studies, we conducted a simple correlation analysis to test this hypothesis (α = 0.05).

### Correlation between baseline hippocampal FC and rTMS-induced changes in FC among hippocampal network nodes

The purpose of this analysis was to determine whether we could reproduce the finding of Wang et al. (2014), that baseline hippocampal FC predicted the degree of PPC rTMS-induced change in hippocampal FC among brain areas. We first found clusters of voxels in our data where rTMS produced a significant increase in hippocampal FC at a threshold of *p* < 0.01, with no spatial extent threshold. Like Wang et al. (2014), we applied a liberal threshold to include a range of change values. This resulted in 183 significant clusters, which we then divided into automated anatomic labeling (AAL; Tzourio-Mazoyer et al., 2002)-defined anatomic regions and all regions with >15 voxels were included in the analysis. The 15-voxel threshold was applied to ensure that each cluster contained enough voxels to calculate a reliable mean time series. This resulted in 95 clusters. We then formed a correlation matrix for each subject and time point by comparing the mean time series of each cluster with that of each other cluster (*3dNetCorr*). Next, we averaged the correlation matrices within each time point across subjects and subtracted the prestimulation correlation matrix from the poststimulation matrix. This resulted in a single matrix, which we sorted by baseline hippocampal FC. Then, to determine whether baseline hippocampal FC predicted the rTMS-induced change in FC, we plotted the baseline hippocampal FC of each cluster against the mean change in FC between that cluster and every other cluster. Finally, to determine whether these changes were specific to FC with the hippocampus, we performed the same analyses, but replaced hippocampal FC with GC for each cluster. Additionally, we re-sorted these matrices by region to reveal, qualitatively, areas where hippocampal nodes and nodes that increased in GC, showed the highest change in FC.

### Statistical analyses

All analyses were conducted using R software. Shapiro–Wilks tests of normality were conducted before each analysis. Table 1 lists the specifications of each test, including critical values, the data used in each test, and confidence intervals. In the Results, an alphabetic code is listed with each test linking it to additional details in Table 1.

**Table 1.** Statistics table indicating the results of all analyses

Manuscript	Figure	Sample	Data type	Data structure	Type of test	Multiple comparison correction	Program	Statistics	*p* values	Confidence intervals
a		Current	Spacing between stimulation sessions	Non-normal distribution	Mann–Whitney (between groups; PPC group: participants receiving 3 vs 4 d of stimulation)		R	*W* = 36	*p* = 0.327	Mean = 0.1092, 95% CI [–0.0743, 0.2110]
b		Current	Average motion displacement	Non-normal distribution	Wilcox rank-sum (all subjects; post vs pre)		R	*V* = 167	*p* = 0.194	Mean = 0.0074 95% CI [–0.0032, 0.0204]
c		Current	Average motion displacement	Non-normal distribution	Mann–Whitney (parietal vs vertex)		R	*W* = 199.5	*p* = 0.350	Mean = –0.0099 95% CI [–0.0225, 0.0096]
d		Current	Number of censored trials	Non-normal distribution	Wilcox rank-sum (all subjects; post vs pre)		R	*V* = 118.5	*p* = 0.155	Mean = 2.5000 95% CI [–0.9999, 6.5000]
e		Current	Number of censored trials	Non-normal distribution	Mann–Whitney (parietal vs vertex)		R	*W* = 218.5	*p* = 0.604	Mean = –2.1121 95% CI [–3.0000, 0.00004]
f	2	Current	Whole-brain FC analysis, retrosplenial cortex	Non-normal distribution	Wilcox rank-sum (within-groups; PPC group, *post hoc* test, post vs pre active stimulation)	Bonferroni	R	*V* = 7	*p* = 1.16 × 10^–3^	Mean = 0.1697, 95% CI [0.0654, 0.2590]
g	2	Current	Whole-brain FC analysis, fusiform gyrus	Non-normal distribution	Wilcox rank-sum (within-groups; PPC group, *post hoc* test, post vs pre active stimulation)	Bonferroni	R	*V* = 4	*p* = 4.27 × 10^–4^	Mean = 0.1475, 95% CI [0.0951, 0.2132]
h	2	Current	Whole-brain FC analysis, lateral PC	Non-normal distribution	Wilcox rank-sum (within-groups; PPC group, *post hoc* test, post vs pre active stimulation)	Bonferroni	R	*V* = 1	*p* = 1.22 × 10^–4^	Mean = 0.1331, 95% CI [0.0777, 0.2034]
i	2	Current	Whole-brain FC analysis, superior PC	Non-normal distribution	Wilcox rank-sum (within-groups; PPC group, *post hoc* test, post vs pre active stimulation)	Bonferroni	R	*V* = 2	*p* = 1.83 × 10^–4^	Mean = 0.1682, 95% CI [0.0815, 0.2294]
j	3*A*	Current	Hippocampal-LPOC FC changes (a priori)	Non-normal distribution	Wilcox rank-sum (within-groups; PPC group, post vs pre active stimulation)		R	*V* = 95	*p* = 0.048	Mean = 0.0867, 95% CI [0.0013, 0.2053]
k	3*A*	Current	Hippocampal-LPOC FC changes (a priori)	Non-normal distribution	Mann–Whitney (between groups; PPC group vs vertex group)		R	*W* = 93	*p* = 0.034	Mean = 0.1367, 95% CI [0.0195, 0.3257]
l	4	Current	Hippocampal-LPOC FC changes	Non-normal distribution	Permutation test (between groups; PPC group vs vertex group)		R	Observed mean difference = 0.1795		95% of distribution [0.1098, 0.2536]
m	3*A*	Current	Hippocampal-LPOC FC changes (a priori)	Non-normal distribution	Wilcox rank-sum (within groups; vertex group, post vs pre active stimulation)		R	*V* = 7	*p* = 0.148	Mean = –0.0477, 95% CI [–0.0554, 0.2357]
n	3*A*	Current	DLPFC-LPOC FC changes (a priori)	Non-normal distribution	Wilcox rank-sum (within groups; PPC group, post vs pre active stimulation)		R	*V* = 81	*p* = 0.252	Mean = 0.0444, 95% CI [–0.0344, 0.1270]
o	3*A*	Current	DLPFC and Hippocampal-LPOC changes (a priori)	Non-normal distribution	Wilcox rank-sum (within groups; PPC group, DLPFC-LPOC vs hippocampal-LPOC FC)		R	*V* = 75	*p* = 0.421	Mean = 0.0344, 95% CI [–0.0636, 0.1593]
p		Current	GC-LPOC changes (a priori)	Non-normal distribution	Wilcox rank-sum (within groups; PPC group, post vs pre active stimulation)		R	*V* = 94	*p* = 0.055	Mean = 0.0179, 95% CI [–0.0006, 0.0434]
q		Current	GC and Hippocampal-FC changes (a priori)	Non-normal distribution	Wilcox rank-sum (within groups; PPC group, post vs pre active stimulation)		R	*V* = 92	*p* = 0.073	Mean = 0.0641, 95% CI [–0.0066, 0.1547]
r		Current	DLPFC and hippocampal target changes (control analysis)	Normally distributed	Paired *t* test (within-groups; PPC group, post vs per active stimulation)		R	*t*_(14)_ = 0.949	*p* = 0.359	Mean = 0.048 95% CI [–0.061, 0.157]
S		Current	DLPFC and stimulus location changes (control analysis)	Non-normal distribution	Wilcox rank-sum (within-groups; PPC group, post vs per active stimulation)		R	*V* = 68	*p* = 0.679	Mean = 0.026 95% CI [–0.103, 0.121]
t		Current	Changes in PPC GC	Non-normal distribution	Wilcox rank-sum (within-groups; PPC group, post vs per active stimulation)		R	*V* = 92	*p* = 0.073	Mean = 0.022 95% CI [–0.005, 0.050]
u	3*B*	Current	Hippocampal FC changes within Yeo Networks	Normally distributed	Paired *t* test (within groups; PPC group, post vs pre active stimulation)		R	*t*_(16)_ = 10.96	*p* = 7.6 × 10^–9^	Mean = 0.0900, 95% CI [0.0725, 0.1073]
v	3*B*	Wang et al. (2014)	Hippocampal FC changes within Yeo networks	Normally distributed	Paired *t* test (within groups; PPC group, post vs pre active stimulation)		R	*t*_(16)_ = 11.27	*p* = 5.10 × 10^–9^	Mean = 0.0169, 95% CI [0.0138, 0.0201]
w	3*B*	Both samples	Hippocampal FC changes within Yeo networks	Normally distributed	Paired *t* test (between groups; current vs Wang, active stimulation)		R	*t*_(32)_ = 8.75	*p* = 5.42 × 10^–10^	95% CI [0.0560, 0.0900]
x	6	Both samples	Hippocampal FC changes within Yeo networks	Non-Normally distributed	Spearman correlation across samples (current and Wang)		R	*r* = 0.51	*p* = 0.037	95% CI [0.0389, 0.7956]
y		Both samples	GC changes within Yeo networks	Non-Normally distributed	Spearman correlation across samples (current and Wang)		R	*r* = 0.16	*p* = 0.536	95% CI [–0.3419, 0.5989]
z	8	Current	Hippocampal FC changes in significant regions (*p* < 0.01)	Non-normal distribution	Spearman correlation (within groups; baseline hippocampal FC and hippocampal-FC changes)		R	*r* = 0.39	*p* = 1.0 × 10^–4^	95% CI [0.2002, 0.5453]
aa		Current	Hippocampal FC changes in significant regions (*p* < 0.01), outlier removed	Normally distributed	Pearson correlation (within groups; baseline hippocampal FC and hippocampal-FC changes)		R	*r*_(92)_ = 0.47	*p* = 1.14 × 10^–6^	95% CI [0.2955, 0.6141]
bb	8	Current	GC changes in significant regions (*p* < 0.01)	Non-normal distribution	Spearman correlation (within groups; baseline GC and GC changes)		R	*r* = –0.08	*p* = 0.39	95% CI [–0.2593, 0.1046]

Each analysis includes a letter indicator (“manuscript” column) linking the test in the table to the analysis in the text. The link to the corresponding figure, if any, and the sample used for the test are indicated in the “figure,” and “sample,” columns, respectively. The “current” sample includes tests using data from the current work, and the previous study is indicated as Wang et al. (2014), the dependent variables for each test are listed as “data type,” and the “data structure” column indicates whether the data are normally distributed. The type of test, contrast, and the groups used for the analysis are listed in the “type of test” column. The multiple correction method is listed under “multiple comparisons correction.” the program used to perform the analysis is included under “program.” The critical value and degrees of freedom are listed for each test under “statistics.” Finally, the *p* value and confidence intervals are listed in the final two columns. DLPFC, dorsolateral prefrontal cortex; GC, global connectedness; LPOC, left precuneus and medial occipital cortex.

## Results

The interval between rTMS sessions was 23.9 ± 3.0 h for the PPC group and 24.3 ± 2.7 h for the vertex group (non-significant; Table 1, a, *W* = 36, *p* = 0.327, 95% CI [–0.0743, 0.2110]). Head motion, calculated as average head frame displacement in six directions, did not significantly differ between scans (prestimulation vs poststimulation; Table 1, b, *V* = 167, *p* = 0.194, 95% CI [–0.0743, 0.2110]) or groups (parietal vs vertex; Table 1, c, *W* = 199.5, *p* = 0.350, 95% CI [–0.0225, 0.0096]). The same was true for the number of censored TRs during denoising (prestimulation vs poststimulation; Table 1, d, *V* = 118.5, *p* = 0.155, 95% CI [–0.9999, 6.5000]; parietal vs vertex; Table 1, e, *W* = 218.5, *p* = 0.604, 95% CI [–3.0000, 0.00004]). Average head displacement was 0.089 ± 0.005 mm per frame. The average number of censored TRs per scan was 5.348 ± 1.561.

**Figure 2.**
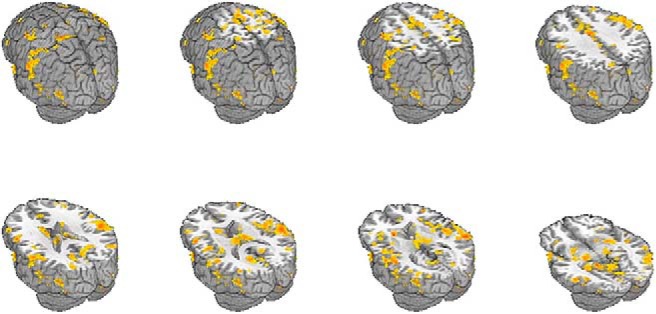
Regions showing significant change in hippocampal FC following PPC rTMS from the current study (FDR corrected, *q* = 0.05).

Figure 2 shows regions that changed in FC with the hippocampus (FDR corrected, *q* = 0.05) in the current sample. These changes were all increases. PPC rTMS produced significant increases in hippocampal FC in all of the areas reported by Wang et al. (2014), including left retrosplenial cortex (Table 1, f, *V* = 7, *p* = 1.16 × 10^−3^, 95% CI [0.0654, 0.2590]), left fusiform gyrus (Table 1, g, *V* = 4, *p* = 4.27 × 10^−4^, 95% CI [0.0951, 0.2132]), left lateral PC (Table 1, h, *V* = 1, *p* = 1.22 × 10^−4^, 95% CI [0.0777, 0.2034]), left superior PC (Table 1, i, *V* = 2, *p* = 1.83 × 10^−4^, 95% CI [0.0815, 0.2294]; all results Bonferroni corrected).

**Figure 3.**
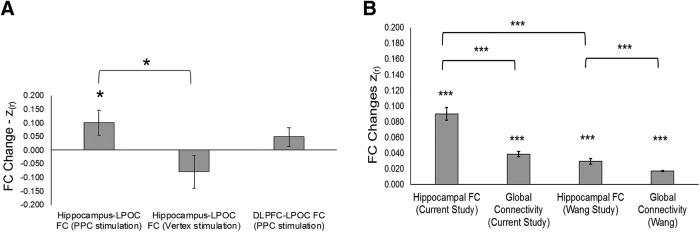
***A***, Average change in hippocampal-LPOC FC for subjects receiving PPC stimulation (left bar) and vertex stimulation (middle bar). Average DLPFC-LPOC FC changes for subjects receiving PPC stimulation is represented by the right bar. ***B***, Mean changes in hippocampal FC within 17 segregated networks from Yeo et al. (2011) after PPC rTMS in this study and Wang et al., and change in GC within networks from both studies. Error bars represent the standard error of the mean; *p < 0.05, ***p < 0.0001.

**Figure 4.**
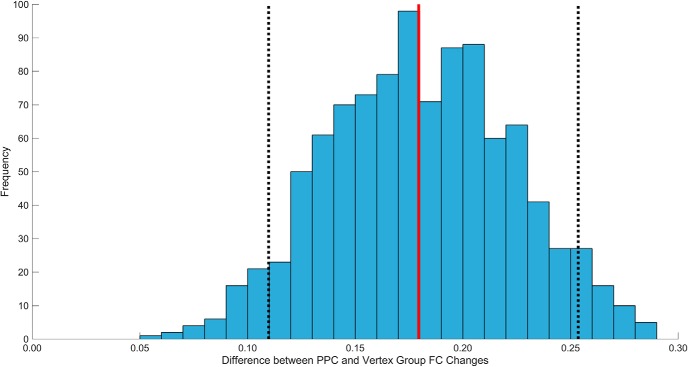
Histogram representing the result of 1000 group mean differences using eight subjects from each group, where the eight PPC subjects are randomly sampled each time. The black dotted lines represent the upper (0.2536) and lower (0.1098) limit of 95% of the distribution. The observed mean difference between the PPC and vertex group is shown by the red line (0.1795).

In our reanalysis of the Wang et al. (2014) data, the LPOC region of interest showed significantly increased FC with the hippocampus after active rTMS, relative to sham. In the current sample, we also found that PPC rTMS caused significant increases there (Table 1, j, *V* = 95, *p* = 0.048, 95% CI [0.0013, 0.2053]; Fig. 3*A*). This increase [z_(r)_ = 0.20 ± 0.04; mean_(SEM)_] was larger than, and opposite in direction to, the mean change after vertex rTMS (z_(r)_ = –0.08 ± 0.06; Fig. 3*A*). The changes in the PPC rTMS group were significantly greater than the changes in the vertex group (Table 1, k, *W* = 93, *p* = 0.034, 95% CI [0.0195, 0.3257]). Vertex stimulation did not cause changes in hippocampal-LPOC FC (Table 1, l, *V* = 7, *p* = 0.148, 95% CI [–0.055, 0.2357]). Resampling the group differences in hippocampal-LPOC FC in 1000 matched groups of eight subjects showed no instances where changes were greater in the vertex group, including those bounded by 95% of the distribution (Table 1, m, observed mean = 0.1795, 95% of distribution [0.02513, 0.03392]; Fig. 4). Thus, it is unlikely that our results were driven by differences in sample size between groups. Whole-brain analyses of hippocampal FC changes in the vertex group did not reveal any significant clusters (all *p* > 0.05). The same was true when measuring FC from the vertex stimulation site.

In the current data, DLPFC-LPOC FC did not increase significantly after PPC rTMS (Table 1, n, *V* = 81, *p* = 0.2524, 95% CI [–0.0344, 0.1270]; Fig. 3*A*), but the DLPFC-LPOC FC change did not differ significantly from the hippocampal-LPOC FC change (Table 1, o, *V* = 75, *p* = 0.4212, 95% CI [–0.0636, 0.1593]), nor did LPOC-GC (Table 1, p, *V* = 94, *p* = 0.055, 95% CI [–0.0006, 0.04139]). However, there was a trend-level difference between the GC and hippocampal FC changes in the LPOC region (Table 1, q, *V* = 92, *p* = 0.073, 95% CI [–0.0066, 0.1547]). We conducted additional control analyses to determine whether stimulation caused significant increases in FC between the DLPFC and the hippocampus, but it did not (Table 1, r, *t*_(14)_ = 0.949, *p* = 0.359, 95% CI [–0.061, 0.157]), nor were there changes in FC between the DLPFC and the stimulus location in the PPC (Table 1, s, *V* = 68, *p* = 0.679, 95% CI [–0.103, 0.121]), nor did PPC-GC increase (Table 1, t, *V* = 92, *p* = 0.073, 95% CI [–0.005, 0.050]).

**Figure 5.**
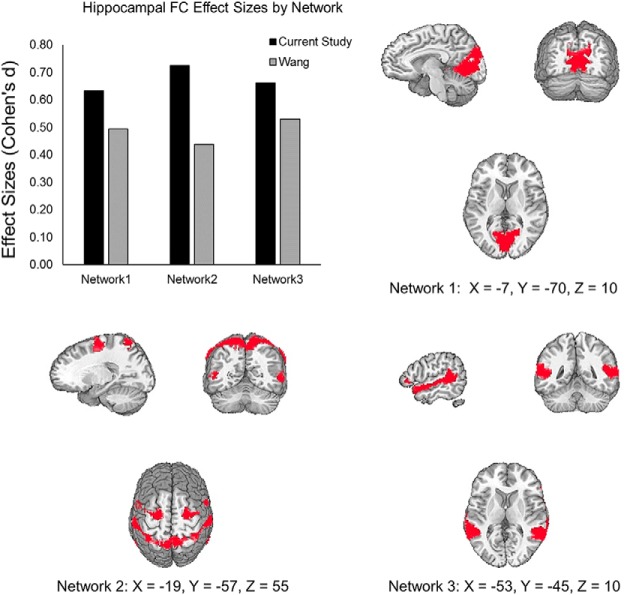
Effect size of increases in hippocampal FC within three representative networks from Yeo et al. (2011) after PPC rTMS in this study and Wang et al. Network 1 includes cuneus and retrosplenial cortex. Network 2 includes somatosensory areas. Network 3 includes superior temporal areas.

In the current sample, there was an increase in hippocampal FC with the 17 networks identified by Yeo et al. (2011), which was significantly stronger than the GC changes in these networks (Table 1, u, *t*_(16)_ = 10.96, 7.6 × 10^−9^, 95% CI [0.0725, 0.1073]). We found the same effect in the data from Wang et al. (Table 1, v, *t*_(16)_ = 11.27, *p* = 5.10 × 10^−9^, 95% CI [0.0138, 0.0201]; Fig. 3*B*). Comparing the hippocampal FC changes between studies, we found that they were larger in the current study, despite using fewer stimulation sessions (Table 1, w, *t*_(32)_ = 8.75, *p* = 5.42 × 10^−10^, 95% CI [0.0560, 0.09]), although GC changes were also larger in the current results than in the Wang et al. data. This suggests overall differences in the magnitude of FC changes across experiments, which could reflect factors such as different scan variables between studies. After PPC stimulation in both studies, increases in hippocampal FC were maximal in networks that included the cuneus and retrosplenial, somatosensory, and superior temporal, areas (Fig. 5).

**Figure 6.**
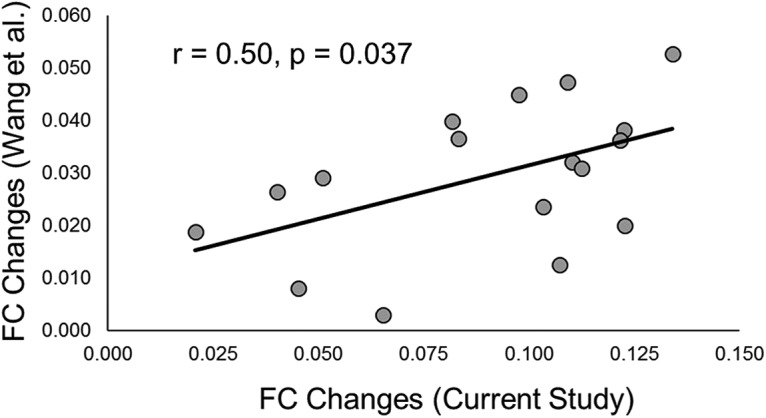
Scatterplot of PPC rTMS-induced hippocampal FC [z_(r)_] changes across networks from Yeo et al. (2011). Each dot represents the rTMS-induced hippocampal FC change from the current study (*x*-axis) and Wang et al. (*y*-axis) within one of the 17 networks from Yeo et al. (2011). The black line represents the regression line across individual data points.

In our test for whether the whole-brain topographic patterns of rTMS-induced hippocampal FC were similar between studies, we found that FC changes were correlated between studies (Table 1, x, *r* = 0.51, *n* = 17, *p* = 0.037, 95% CI [0.0389, 0.7956]; Fig. 6). There was no significant correlation between the GC changes in the current study with the hippocampal FC changes of Wang et al. (Table 1, y, *r* = 0.16, *n* = 17, *p* = 0.536, 95% CI [–0.3419, 0.5989]). These results indicate that the magnitude of hippocampal FC changes across networks was similar between studies and that their topographic distribution was reproducible.

**Figure 7.**
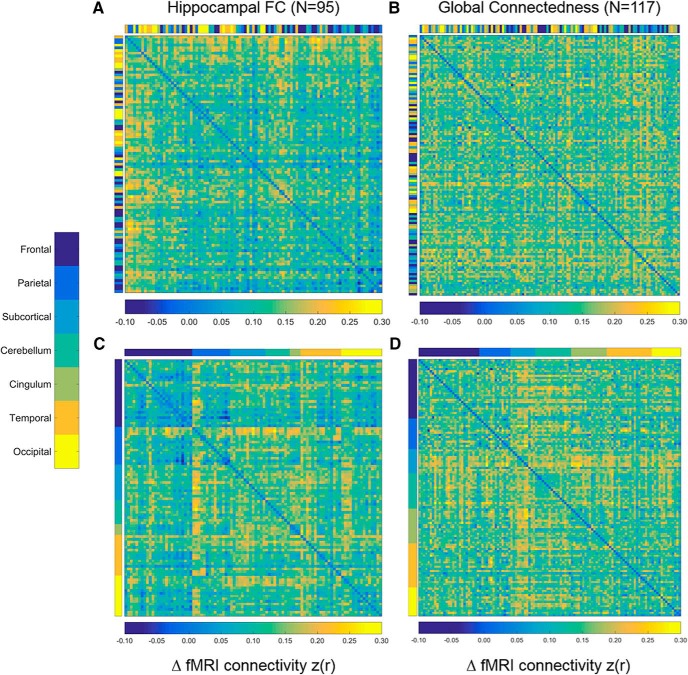
Correlation matrices of regions demonstrating significant (*p* < 0.01) changes in hippocampal (***A***) and global (***B***) FC. Matrices are sorted by baseline FC with the highest values represented at the top of the matrices on the *y*-axis and to the left on the *x*-axis. Color bars aligned with each axis represent AAL-defined regions. ***C***, ***D***, Identical to ***A***, ***B*** but are sorted by region.

**Figure 8.**
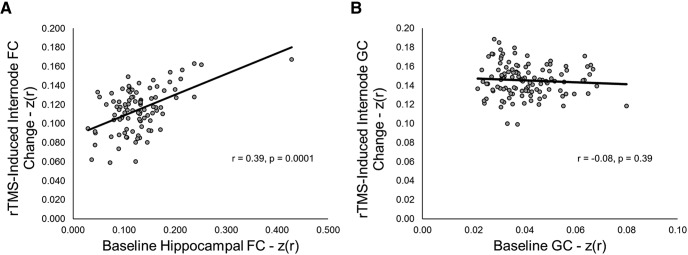
***A***, Scatterplot of baseline hippocampal FC for regions demonstrating significant (*p* < 0.01) changes in hippocampal FC and average rTMS-induced FC change in those regions. ***B***, Scatterplot of baseline GC for regions demonstrating significant (*p* < 0.01) changes in GC and average rTMS-induced internode GC change in those regions.

Finally, we reproduced the finding that, among areas showing significant increases in hippocampal FC after PPC rTMS, prestimulation hippocampal FC predicted the magnitude of the increase (Fig. 7*A*). This was confirmed by the relationship between the baseline and mean change in hippocampal FC across areas (Table 1, z, *r* = 0.39, *n* = 95, *p* = 1.0 × 10^−4^, 95% CI [0.2002, 0.5453]; Fig. 8*A*). Removing the single outlier did not change the significance of the correlation (Table 1, aa, r_(92)_ = 0.47, *p* = 1.14 × 10^−6^, 95% CI [0.2955, 0.6141]). We did not observe the same pattern of results when performing the same analyses using GC as the dependent variable (Table 1, bb, *r* = –0.08, *n* = 115, *p* = 0.39, 95% CI [–0.2593, 0.1046]; Figs. 7*B*, 8*B*). These findings indicate a specific effect of PPC rTMS on the hippocampus and rule out non-specific enhancement of FC across the brain. Re-sorting the matrices in Figure 7 revealed no regional differences in the change in GC (Fig. 7*B*), but did show that, among regions connected to the hippocampus at baseline, frontal regions showed qualitatively less change than more posterior regions, such as the PPC, similar to the results of Wang et al. (2014) and consistent with the interpretation that areas with higher baseline FC with the hippocampus change most with PPC rTMS.

## Discussion

We independently reproduced the highly specific increase in hippocampal FC, reported by Wang et al. (2014), resulting from high-frequency rTMS of PPC, using a partial replication of their technique and adding additional new controls. We applied a whole-brain analysis as well as a hypothesis-based approach, predicated on the anatomic distribution of changes reported by Wang et al. (2014). We also looked for changes in hippocampal FC within 17 additional segregated brain networks (Yeo et al., 2011). The whole-brain comparison to Wang et al. (2014) revealed that PPC rTMS caused significant hippocampal FC changes in all of the regions reported by Wang et al., as well as several others. The hypothesis-based approach revealed significant increases in hippocampal FC with the LPOC, a region derived from our re-analysis of the Wang et al. (2014) data. These changes were specific to FC with the hippocampus: PPC rTMS did not significantly increase FC between the DLPFC, an area active in many cognitive processes including learning, and the LPOC. We also ruled out the possibility that the findings reflected a general increase in brain connectivity: Hippocampal FC was significantly greater than GC across all networks examined in both the present and the Wang et al. data. Although our vertex control sample was small, we found no significant FC changes in this group and hippocampus-LPOC FC was significantly greater for the PPC rTMS group than the vertex group.

As in the data of Wang et al. (2014), baseline hippocampal FC predicted PPC rTMS-induced FC changes and we demonstrated the specificity of this relationship by showing that baseline GC did not predict GC increases after rTMS. Finally, the spatial pattern of rTMS-induced FC change was similar and correlated between studies. Taken together, this is strong evidence that the effect of 20-Hz rTMS on the PPC on hippocampal FC is robust, reproducible, and highly specific in anatomic terms.

Notably, we were able to reproduce, and possibly to exceed, the results of Wang et al. (2014) with fewer stimulation sessions. Multiple consecutive rTMS sessions are burdensome to subjects and investigators alike and reducing the requirement increases the attractiveness of the PPC rTMS paradigm.

Our vertex rTMS group showed decreased hippocampal FC with almost every network, including the LPOC. This unexplained time-related drift could be due to a physiologic effect and might represent a potential confound. However, as noted above, others (Jung et al., 2016) have found no evidence of FC changes from vertex rTMS. Additionally, the average change in hippocampal FC across the networks from Yeo et al. (2011) in the Wang et al. (2014) sham data did not differ significantly from zero.

There were several procedural differences between the current work and that of Wang et al. (2014), the most obvious of which was the absence of behavioral testing. Therefore, we do not know whether the changes in hippocampal FC were associated with an improvement in declarative memory. Additionally, there were differences in how we preprocessed our resting-state data. We did not bandpass filter our data and, unlike Wang et al. (2014), we included spatial smoothing to reduce the influence of spatial noise and increase signal-to-noise ratio (SNR).

Another difference between studies was our use of a vertex stimulation control. Wang et al. (2014) used subthreshold stimulation (10% of RMT) of the PPC as their within-subjects control, which may have caused weak local brain effects without reproducing the somatosensory effect of full-intensity rTMS. They also used full-intensity stimulation of the motor cortex in an independent group as a secondary control, and this was all but certain to produce widespread changes in FC. We chose active-intensity stimulation at the vertex as our control because others (Jung et al., 2016) found no effect on FC from stimulation there. This site lies over the sagittal sinus and the interhemispheric fissure, where the cortex is relatively distant from the coil and the nearest regions are out of the plane of the stimulating current. Neither in this, nor the study of Wang et al. (2014), did control stimulation produce any measurable increase in hippocampal FC.

Finally, for reasons described above, we used three and, in some cases, 4 d of rTMS, while Wang et al. (2014) used five. This study contains no basis for a quantitative comparison of the strength or duration of the connectivity or behavioral changes produced by various treatment durations, but 3–4 d appears adequate to produce FC changes similar to those of Wang et al. (2014) at a 24-h delay. These procedural differences do not allow us to claim a strict replication of the paradigm, but they do not detract from the substantial reproduction of the result and could not have caused it by themselves.

Both we and Wang et al. (2014) were able to produce dramatic increases in hippocampal network FC with a few sessions of PPC rTMS, making this one of the strongest and most reliable effects in noninvasive neuromodulation. The differences in the treatment paradigms and image processing procedures decrease the likelihood that both studies arrived at a similar result due to an artifact or systematic noise. Others (van der Werf et al., 2010; Vercammen et al., 2010; Gratton et al., 2013; Rahnev et al., 2013; Steel et al., 2016; Rastogi et al., 2017) have also used FC to study how rTMS affects brain function at the network level. FC in the default mode network appears to be particularly sensitive to modulation with rTMS (Eldaief et al., 2011; Halko et al., 2014; Wang et al., 2014) and can be modulated by stimulating the PPC (Eldaief et al., 2011; Wang et al., 2014) and the cerebellum (Halko et al., 2014). The latter study also used individual FC to choose the stimulation target.

Future studies may consider examining whether the stimulation regimen itself, largely inspired by conventional rTMS treatment for depression (George et al., 1997), where it was adopted without systematic exploration of the parameter space, is optimal, and whether even more dramatic or faster responses are attainable using optimized stimulation parameter settings.

## Conclusion

The hippocampal network FC changes reported by Wang et al. (2014) after PPC rTMS are reproducible in magnitude, specificity, and topographic distribution. Our additional analyses, ruling out changes in global correlation, further strengthen the evidence for the selectivity of this approach for the hippocampal network. Moreover, our findings suggest that these effects are achievable with fewer than five stimulation sessions. This provides encouraging support for PPC rTMS as a means of enhancing memory network FC and for rTMS in general as a technique for producing targeted changes in brain network connectivity.

## Synthesis

Reviewing Editor: Tatiana Pasternak, University of Rochester Decisions are customarily a result of the Reviewing Editor and the peer reviewers coming together and discussing their recommendations until a consensus is reached. When revisions are invited, a fact-based synthesis statement explaining their decision and outlining what is needed to prepare a revision will be listed below. The following reviewer(s) agreed to reveal their identity: Greg Applebaum.

The reviewers welcomed the study and commented on the importance of replication, particularly since the work is the reassessment of the results of an earlier impactful TMS study which showed changes in functional connectivity in the hippocampal network. While the reviewers listed a number of important strengths, they also raised a number of reservations that must be addressed before the manuscript can be considered for publication in eNeuro.

These are summarized below and are listed in greater detail in the two reviews.

1. Both reviewers pointed out that some of the methodology was changed and the control conditions deviated from that in the original study, making the label “replication” not strictly accurate. Please, address and motivate your choice of control condition and discuss potential impact of the differences in the control conditions between the current and the previous study.

2. While the original study included behavioral assessment of the TMS-induced change in functional connectivity, such assessment was not included in this manuscript. The lack of behavioral data was considered to be a problem. Please address it.

3. Reviewer 1 lists a number of missing methodological details, including rTMS parameters, spacing across sessions, etc. and has suggestions for Figs 4 and 5. In addition, the reasons for the differences between numbers of sessions for different subjects should be provided.

4. Please provide rationale for multiple seed regions and include an image showing their locations

5. Please cite the relevant work from other groups which successfully combined rTMS and fMRI, listed by reviewer 1.

6. Please provide the caption for the Table that will provide readers with guidance how to read it.

REVIEWER 1.

ADVANCES IN THE FIELD

Replication - but only partial replication as behavioural data is not included

COMMENTS TO THE AUTHORS

It is great to see this replication study by an independent group. It is a bit disappointing that some of the methodology was changed part way (number of sessions) and method for choice of stimulation site, but on the other hand reassuring that replication ensued regardless of such details. However, one of the most central and impressive elements of the original study was that changes in FC translated into behavioural / memory task performance. This would appear to be a critical element to include in a replication study and the absence of these measures here reduces the impact of this replication study.

Could the authors specify whether the rTMS parameters were identical to those used previously (it is implied that they are)? Was the spacing across sessions similar across subgroups? In the "procedures" section it could be helpful to in brackets refer/guide the reader to the rTMS section.

Why did the authors give 3 daily sessions to 9 individuals and 4 to 6 individuals? What was the spacing? Were these sessions repeated across days? How does this compare to the original study?

It is interesting that Voss and colleagues tried to replicate their own experiment and I believe their most recent update suggested that they could only achieve these results with sessions across a minimum of 5 days. To be fair, this work is only in conference abstract form so far and preliminary. The earlier abstract states that a minimum of 4 days was necessary. The authors are presumably aware of this work by their collaborators. If there are any factors that the authors can identify that might lead to earlier changes, this could be worth including in their paper.

https://abstractsonline.com/pp8/#!/4649/presentation/33453

I found it odd that the authors did not apply band pass filtering - this is a standard and important step to remove non-physiological noise. In a sense, it is intended to remove DoF and the author's argument about retaining DoF seems unusual.

What was the rationale for using multiple seed regions (and choosing the most connected site)? Would this not alter the target region? Should this not impact task performance and the capacity to modify connectivity/performance related to a specific target? It would be useful to include an image that depicts the location of these seed regions. This might be possible with a semi-transparent visualisation of the hippocampus. Could the authors expand on why they switched to sampling 97 preselected regions and how these were identified a priori.

-I may be mistaken, but the data driven approach seemed somewhat circular in parts and likely to firstly increase statistical significance and secondly constrain multiple comparisons to increase the propensity for replication in specific regions:

-For the section "Data-driven, voxel-wise, changes in hippocampal network FC across the brain", although a mask was generated to exclude white matter and CSF, it appears that the subsequent analysis involved considering only clusters within regions previously identified by Wang with a Bonferoni corrected significance threshold of 0.05/4. Is this correct? I think it might be possible to state this more clearly in the text, perhaps with the aid of a flowchart figure for this method and the one below.

-Similarly, in the section "Hypothesis-based comparison to the Wang et al. results", generating a mask by subtracting pre from post-stim data on a subject by subject basis to identify a cluster and then run stats on this mask appears likely to promote statistical significance in subsequent tests.

-Global connectedness appears to apply essentially to the FC of the LPOC area with every other voxel in the brain. I think it might be clearer to rename this as LPOC-GC.

-Could the authors include analysis of DLPFC to Hippocampus connectivity, DLPFC to PPC connectivity and PPC GC? I think these would be important and informative control measures. Analysis of LPOC connectivity is important, but these regions are also primary areas of interest.

-Was the magnitude of FC changes from hippocampus to the 17 networks correlated across these 17 networks? This question obviously differs to the analysis presented.

-Could the authors expand on any changes elicited by vertex stim (i.e. in its functionally connected regions)?

-Having a smaller sample size for vertex stim reduces the power to detect significant changes and thereby reduces the validity of this control. Sample size for vertex stim should be increased to match that of PPC stim.

-The authors state that "the paradigm of combined rTMS and fMRI for individualized targeting and analysis has not been applied to other stimulation sites and networks and it is impossible to predict whether it will be as effective in other systems." Actually, related approaches have been tried in other systems with success (Eldaief et al., PNAS, 2011; Halko et al., J Neuroscience, 2014; Rastogi et al., Neuroimage, 2017), with Halko utilizing individualized targeting. This work by other groups could be acknowledged at this point.

-Fig 4 - it would be useful to include the r/p values

-Fig 5 - I am not sure if this would work, but it could be useful to sort the indices by region for easier interpretation. The categorical colour bar doesn't help that much in my view.

REVIEWER 2

ADVANCES IN THE FIELD:

Replication studies are essential to the growth of neurostimulation. In particular, replication studies of highly impactful findings, such as Wang et al advance the field towards the application of these findings to improve mental health.

COMMENTS TO THE AUTHORS

Overview

The present study is an independent replication of Wang et al., 2014. In the original Wang et al study, it was reported that that five daily sessions of repetitive transcranial magnetic stimulation to the posterior parietal cortex (PPC) produced significant increases in functional connectivity (FC) in a network centered on the hippocampus, and a correlated increase in memory performance (though the memory improvement versus sham wasn't significant). In the current replication study, the authors performed a very similar paradigm to Wang et al., using similar targeting procedures and stimulation parameter values, but fewer stimulation sessions (namely, two groups with 3 or 4 days of intervention). The current paradigm also used a vertex control condition, rather than 10% rMT, as was used in Wang et al. and conducted several additional analyses, for example using canonical brain networks from Yeo et al 2011. The current study also reprocessed and reanalyzed data from Wang et al. to make direct comparisons between the 2 studies.

Results indicated that they were able to closely replicate the same pattern of findings from Wang. In particular, they observed that PPC rTMS caused significant hippocampal FC changes in all of the regions reported by Wang et al., as well as several others, including significant increases in hippocampal FC with the LPOC, a region derived from their re-analysis of the Wang et al. data. These changes were specific to FC with the hippocampus and the authors were able to rule out the possibility that these findings reflected a general increase in brain connectivity as the hippocampal FC was significantly greater than changes in global connectivity. For their vertex control they found no significant FC changes, though quantitatively this group produced an opposite pattern of FC decreases with the Hippocampus. Importantly, though, hippocampus-LPOC FC was significantly greater for the PPC rTMS group than the vertex group, indicating specificity for the planned hypothesis test.

Review

Human neurostimulation research has grown rapidly in frequency and scope with a number of notable findings that speak to the potential mental health benefits of the approach. Findings such as those reported in Wang et al, in particular, hold great promise for improving healthy and abnormal age-related memory declines. Nonetheless, despite the frequent occurrence of false positives in neurostimulation research, replication studies are far to infrequent. As the Wang et al study is an important and potentially impactful study, the current replication study would make a welcome addition to the growing neurostimulation literature.

Overall, this manuscript was well-written and concise, with clearly articulated motivation and sensible findings and conclusions. The methods are strong and the analyses appropriate, making this an internally valid study. Among the interesting findings are the specificity of the hippocampal FC changes, and the observation that they can be obtained after only four treatment session.

My greatest concern with this study is that it uses a fundamentally different control condition relative to Wang et al, which technically means that it is not a replication study. In Wang et al, the control condition was derived from stimulation at 10% of resting motor threshold, whereas here control was versus 100% rMT at vertex. This difference could have profound and meaningful consequences and certainly deserved much greater elaboration in the manuscript. I'd suggest both motivating this difference in the introduction/methods to address why a different control was used AND provide much more content in the discussion about how this could have impacted the findings vis-a-vis Wang et al. Fundamentally, I feel that the selection of a vertex control condition is an improvement in the study design, and this may have contributed to the larger effects obtained in short time, however, it is a highly meaningful difference when attempting to couch this study as a straight replication study. Therefore, more justification and discussion is warranted.

Other minor points...

The table is referenced often in the paper, but it isn't explained how to read the table, nor is a caption provided. Because it is a big, complex table, more introduction and curation of the content is needed.

There are other minor methodological issues, such as difference in smoothing between the studies, and the fact that this study switched FC approaches in mid study... however, I do not think that there are meaningful, or that they need further elaboration in the manuscript.

## Author Response

Dear Dr. Pasternak,

Thank you for your letter and the helpful comments and recommendations of the two reviewers. We have revised the paper and hope you and the reviewers will find it significantly improved.

1. Both reviewers pointed out that some of the methodology was changed and the control conditions deviated from that in the original study, making the label “replication” not strictly accurate. Please, address and motivate your choice of control condition and discuss potential impact of the differences in the control conditions between the current and the previous study.

We agree and now explain the differences between studies in the Methods section, including our rationale for these changes, the use of vertex control, and changes we made to the seeding technique. We have also added text to the Discussion emphasizing these differences and their potential role in shaping our results.

2. While the original study included behavioral assessment of the TMS-induced change in functional connectivity, such assessment was not included in this manuscript. The lack of behavioral data was considered to be a problem. Please address it.

We have changed the title accordingly, and mention this difference in the Introduction (page 4; Tracked changes version) and Discussion (page 19).

3. Reviewer 1 lists a number of missing methodological details, including rTMS parameters, spacing across sessions, etc. and has suggestions for Figs 4 and 5. In addition, the reasons for the differences between numbers of sessions for different subjects should be provided.

All requested details are now presented with greater clarity in the Methods. Figures 4 (now 6) and 6 (now 8) now include r and p-values. We also include the resorted correlation matrix appended to Figure 5 (now 7).

4. Please provide rationale for multiple seed regions and include an image showing their locations

We now include a rationale for our different seed selection techniques on page 8, and a new figure (Figure 1) demonstrates the locations of all subjects' seeds.

5. Please cite the relevant work from other groups which successfully combined rTMS and fMRI, listed by reviewer 1.

We thank Reviewer 1 for bringing this work to our attention. We now include a discussion of these studies on page 20.

6. Please provide the caption for the Table that will provide readers with guidance how to read it.

We thank the reviewer for this suggestion. A detailed description of all table columns is now included for Table 1 as a caption (page 27).

REVIEWER 1

It is great to see this replication study by an independent group. It is a bit disappointing that some of the methodology was changed part way (number of sessions) and method for choice of stimulation site, but on the other hand reassuring that replication ensued regardless of such details.

However, one of the most central and impressive elements of the original study was that changes in FC translated into behavioural / memory task performance. This would appear to be a critical element to include in a replication study and the absence of these measures here reduces the impact of this replication study.

While the seeding technique was changed in our final three subjects receiving PPC stimulation, we gave some participants four sessions of stimulation as part of a dose-finding study. We statistically compared FC changes for participants receiving 3 and 4 days of stimulation using a Wilcox sign rank test, which revealed no significant difference (page 5). Therefore, we decided to pool the 3 and 4-day treatment subjects for this purpose. We have included this rationale on page 5 of the Methods as well as the results of a statistical comparison between participants who received 3 and 4 days of stimulation.

The change to the seeding technique was made to sample a larger area of the hippocampus. We now provide a rationale for both the deviation from Wang et al., and the seeding technique change on page 8 of the Methods.

We agree that a full replication would include memory testing. We have thus changed the title to reflect this difference and emphasize the > 24 hour duration of the effect on connectivity. We also now mention this difference in the Discussion (page 19).

Could the authors specify whether the rTMS parameters were identical to those used previously (it is implied that they are)?

We used the same rTMS parameter values as the previous work. This is now specified on page 9 of the Methods.

Was the spacing across sessions similar across subgroups?

The interval between sessions was 23.86 {plus minus} 3.00 hours for the PPC group and 24.27 {plus minus} 2.69 hours for the vertex group. This difference was not statistically significant. We mention this in the Results (page 14).

In the “procedures” section it could be helpful to in brackets refer/guide the reader to the rTMS section.

We have added a parenthetical on page 5 to direct the reader to this section.

Why did the authors give 3 daily sessions to 9 individuals and 4 to 6 individuals?

As mentioned above, these individuals were studied as part of a dose-finding study using cohorts of three subjects. We found no significant differences in response between the subjects treated at three and four day, so we pooled them. We now mention this along with the corresponding statistical test in the Methods (page 5).

What was the spacing?

As mentioned above, the spacing was approximately 24 hours.

Were these sessions repeated across days?

As we state on page 5: “Nine subjects received three consecutive daily sessions of rTMS delivered to the PPC, six received four daily PPC sessions, and eight received three daily sessions of identical rTMS delivered to the vertex”

How does this compare to the original study?

In Wang et al., (2014) participants received 5 consecutive daily sessions of stimulation and this is now mentioned on page 5.

It is interesting that Voss and colleagues tried to replicate their own experiment and I believe their most recent update suggested that they could only achieve these results with sessions across a minimum of 5 days. To be fair, this work is only in conference abstract form so far and preliminary. The earlier abstract states that a minimum of 4 days was necessary. The authors are presumably aware of this work by their collaborators. If there are any factors that the authors can identify that might lead to earlier changes, this could be worth including in their paper.

https://abstractsonline.com/pp8/#!/4649/presentation/33453

That abstract, although using similar methodology to Wang et al., was not an attempted replication, but a dose-finding study to determine how many sessions of active rTMS are necessary to induce a conservative level of functional connectivity change. The findings in the poster that is mentioned indicated that 4-5 days were needed to reach that threshold, which is a different question than whether fewer days significantly affects functional connectivity. The present methods and findings are therefore not in conflict with the findings described in the cited abstract.

I found it odd that the authors did not apply band pass filtering - this is a standard and important step to remove non-physiological noise. In a sense, it is intended to remove DoF and the author's argument about retaining DoF seems unusual.

Bandpass filtering comes at the cost of reduced degrees of freedom when using the general linear model to estimate and remove nuisance signals and is not universally used in resting-state fMRI connectivity analysis. For example, with a 2-second TR and a bandpass range of 0.001 to 0.08, 66% of the degrees of freedom are removed. In our sample, the combination of motion censoring and bandpass filtering would have reduced our statistical power so greatly that we would have had to eliminate most of our subjects.

Bandpass filtering is also problematic for several reasons. First, as we now state on page 7, physiological noise, which bandpass filtering is intended to remove, is likely to persist in the data, since the respiration (~0.3 Hz) and cardiac (~0.9-1 Hz) frequencies are aliased to frequencies below the Nyquist frequency (Gotts et al., 2013). Second, test-retest reliability is reduced by applying band-pass filtering. Shirer et al. (2015) examined the impact of various preprocessing steps on 3 different data quality measures, including test-retest reliability. Virtually all measures benefit from not applying band-pass filtering, with continuous improvement as the high-frequency cutoff is raised (the best condition is no high cutoff at all). For these reasons, investigators are now beginning to omit this step from their pipelines (e.g. Stoddard et al., 2016; Song et al., 2015; Gotts et al. 2013). Thus, we also elected to omit it as well. We now include this more extensive rationale for omitting bandpass filtering on page 7.

What was the rationale for using multiple seed regions (and choosing the most connected site)? Would this not alter the target region? Should this not impact task performance and the capacity to modify connectivity/performance related to a specific target?

The use of multiple seed regions and selecting the hippocampal seed and PPC target that were maximally connected at baseline were central aspects of the Wang et al. approach and we felt it was important to include them. To make this clear, we have added a description of the Wang et al. method in the “rTMS Targeting” section on page 7.

It would be useful to include an image that depicts the location of these seed regions. This might be possible with a semi-transparent visualisation of the hippocampus. Could the authors expand on why they switched to sampling 97 preselected regions and how these were identified a priori.

We thank the reviewer for this useful suggestion. We have included a figure (Figure 1) with 2 mm radius seeds presented on a template brain.

The decision to change our seeding technique was made to sample a larger area of the hippocampus, as the original method only sampled six points along its longitudinal axis. We have added this rationale on page 8. The 97 voxels included those in the hippocampus within a 15 mm radius of the MNI coordinate identified in Wang et al. (2014; x = -24, y = -18, z = -18). This is now stated on page 8.

-I may be mistaken, but the data driven approach seemed somewhat circular in parts and likely to firstly increase statistical significance and secondly constrain multiple comparisons to increase the propensity for replication in specific regions:

-For the section “Data-driven, voxel-wise, changes in hippocampal network FC across the brain”, although a mask was generated to exclude white matter and CSF, it appears that the subsequent analysis involved considering only clusters within regions previously identified by Wang with a Bonferoni corrected significance threshold of 0.05/4. Is this correct? I think it might be possible to state this more clearly in the text, perhaps with the aid of a flowchart figure for this method and the one below.

We have removed the term “data-driven,” since the analysis was based on a direct comparison to Wang et al. (2014), and thus constrained by a hypothesis. In the Methods, we have changed the name of this section to “Whole brain changes in hippocampal network FC and comparison to Wang et al. (2014).”

-Similarly, in the section “Hypothesis-based comparison to the Wang et al. results”, generating a mask by subtracting pre from post-stim data on a subject by subject basis to identify a cluster and then run stats on this mask appears likely to promote statistical significance in subsequent tests.

We apologize for the confusion here. We created our mask from the results of a reanalysis of Wang et al. and then computed the post-minus-pre difference in FC using the data from the current work within that mask. To make this clear, we have made changes to this section (pages 10-11).

-Global connectedness appears to apply essentially to the FC of the LPOC area with every other voxel in the brain. I think it might be clearer to rename this as LPOC-GC.

The term is also used in our discussion of the Yeo network changes. However, in areas of the text which discuss global connectedness between the LPOC and the rest of the brain, we now apply this shorthand.

-Could the authors include analysis of DLPFC to Hippocampus connectivity, DLPFC to PPC connectivity and PPC GC? I think these would be important and informative control measures. Analysis of LPOC connectivity is important, but these regions are also primary areas of interest.

We agree that these are appropriate control analyses to test for non-specific network effects, and they yielded non-significant results. We have added them to the Results (page 16) and Table 1.

-Was the magnitude of FC changes from hippocampus to the 17 networks correlated across these 17 networks? This question obviously differs to the analysis presented.

We do not understand the relevance of this question to our current analyses and about the best way to quantify such relationships while controlling for the multiple pairwise comparisons that would be necessary. Our analyses indicate that the magnitude of changes in hippocampal FC amongst networks are correlated across studies (page 17) and that increases in hippocampal-cortical FC within the Yeo networks are greater than global connectedness for both studies (page 16). These results are both unrelated to cross-network correlations within each study.

-Could the authors expand on any changes elicited by vertex stim (i.e. in its functionally connected regions)?

We performed a whole-brain analysis of FC changes after vertex stimulation, both from the hippocampus, and from the point of stimulation at the vertex, and applied a liberal threshold of p = 0.05. With the exception of an increase in the medial prefrontal cortex, the hippocampal FC changes, if any, are decreases. No clusters met the threshold for statistical significance for connectivity from the hippocampus or the vertex stimulation point. We now report this in the Results on pages 15-16.

-Having a smaller sample size for vertex stim reduces the power to detect significant changes and thereby reduces the validity of this control. Sample size for vertex stim should be increased to match that of PPC stim.

We agree that the differences in sample sizes may reduce our power to detect changes in the vertex group. However, as mentioned above, even at liberal thresholds, the vertex group did not show any significant increases in FC and showed trends for decreases in many areas. To address the statistical limitation that the reviewer suggests, we conducted a permutation test to determine whether changes for the real/parietal stimulation group would remain significantly different than for the vertex stimulation control group given matched sample sizes for the hypothesis-based analysis (Hipp-LPOC changes). This was performed by subtracting the mean FC change of the 8 vertex subjects from 8 randomly sampled PPC subjects. This process was then repeated 1,000 times to create a distribution of possible group mean differences (see below). The figure shows that all possible combinations yield above 0 differences between groups, with our observed mean difference 0.1795; (red line), and the lower bound of 95% of the distribution (0.10; left dotted line) well above 0 as well. This shows that differences in sample size did not drive our effect.

-The authors state that “the paradigm of combined rTMS and fMRI for individualized targeting and analysis has not been applied to other stimulation sites and networks and it is impossible to predict whether it will be as effective in other systems.” Actually, related approaches have been tried in other systems with success (Eldaief et al., PNAS, 2011; Halko et al., J Neuroscience, 2014; Rastogi et al., Neuroimage, 2017), with Halko utilizing individualized targeting. This work by other groups could be acknowledged at this point.

We now acknowledge these informative studies in the Discussion on page 20.

-Fig 4 - it would be useful to include the r/p values

We have added r and p-values to this figure, and Figure 8.

-Fig 5 - I am not sure if this would work, but it could be useful to sort the indices by region for easier interpretation. The categorical colour bar doesn't help that much in my view.

We performed the regional sorting analysis (see below). In the figure, the top two panels are the original correlation matrices from the initial submission and the bottom left (hippocampal FC changes) and right (GC changes) matrices are sorted by region. While changes in regional differentiation for the GC analysis are not apparent, the hippocampal analysis revealed that frontal regions were much less affected by stimulation than posterior regions, such as the parietal cortex. Baseline connectivity between the hippocampus and a given region remains the most significant predictor of the change in connectivity due to stimulation. These results are consistent with Wang et al. who noticed significant changes in more posterior aspects of the brain, particularly for those areas with high baseline connectivity with the hippocampus. We now mention this on page 17. Additionally, the color scheme of these figures has been changed to accommodate color-blind readers.

REVIEWER 2

My greatest concern with this study is that it uses a fundamentally different control condition relative to Wang et al, which technically means that it is not a replication study. In Wang et al, the control condition was derived from stimulation at 10% of resting motor threshold, whereas here control was versus 100% rMT at vertex. This difference could have profound and meaningful consequences and certainly deserved much greater elaboration in the manuscript. I'd suggest both motivating this difference in the introduction/methods to address why a different control was used AND provide much more content in the discussion about how this could have impacted the findings vis-à-vis Wang et al. Fundamentally, I feel that the selection of a vertex control condition is an improvement in the study design, and this may have contributed to the larger effects obtained in short time, however, it is a highly meaningful difference when attempting to couch this study as a straight replication study. Therefore, more justification and discussion is warranted.

We thank the reviewer for their assessment of our work. This is an important difference between studies, and we agree that it should be emphasized. However, it should be noted that the Wang et al. study included two control conditions: the low-intensity stimulation condition that the reviewer mentions as well as full-intensity stimulation of the motor cortex in an independent group. The second control condition in Wang et al. is similar to that used here, but with vertex as the active control site instead of motor cortex. The rationale for vertex stimulation as our control is now included on page 5, and we acknowledge this difference, and how it potentially affected our results, in the Discussion (pages 19-20).

Other minor points...

The table is referenced often in the paper, but it isn't explained how to read the table, nor is a caption provided. Because it is a big, complex table, more introduction and curation of the content is needed.

We agree and have added the caption (page 27). We also provide an introduction of the table at the end of the Methods section.

There are other minor methodological issues, such as difference in smoothing between the studies, and the fact that this study switched FC approaches in mid study... however, I do not think that there are meaningful, or that they need further elaboration in the manuscript.

We now point out the key differences between studies, for example, our seeding techniques and our control condition. These changes are highlighted throughout the Methods and Discussion.

Very respectfully,

Michael Freedberg, PhD

Postdoctoral Fellow, NINDS/BNU
